# Dissolved Organic Carbon in the North Atlantic Meridional Overturning Circulation

**DOI:** 10.1038/srep26931

**Published:** 2016-05-31

**Authors:** Marcos Fontela, Maribel I. García-Ibáñez, Dennis A. Hansell, Herlé Mercier, Fiz F. Pérez

**Affiliations:** 1 Instituto de Investigaciones Marinas, IIM-CSIC, 36208 Vigo, Spain; 2 Rosenstiel School of Marine and Atmospheric Science, RSMAS/OCE University of Miami, Miami, Florida, USA; 3 CNRS, Laboratoire de Physique des Océans, UMR 6523 CNRS/Ifremer/IRD/UBO, Ifremer Centre de Brest, Plouzané, France

## Abstract

The quantitative role of the Atlantic Meridional Overturning Circulation (AMOC) in dissolved organic carbon (DOC) export is evaluated by combining DOC measurements with observed water mass transports. In the eastern subpolar North Atlantic, both upper and lower limbs of the AMOC transport high-DOC waters. Deep water formation that connects the two limbs of the AMOC results in a high downward export of non-refractory DOC (197 Tg-C·yr^−1^). Subsequent remineralization in the lower limb of the AMOC, between subpolar and subtropical latitudes, consumes 72% of the DOC exported by the whole Atlantic Ocean. The contribution of DOC to the carbon sequestration in the North Atlantic Ocean (62 Tg-C·yr^−1^) is considerable and represents almost a third of the atmospheric CO_2_ uptake in the region.

The Atlantic Meridional Overturning Circulation (AMOC) plays an active role in the cycling and storage of chemical species in the ocean^1^ because it is an entrance portal for chemical tracers into the deep ocean due to water mass formation processes. In the North Atlantic, warm northward flowing surface waters cool and gain density due to strong air-sea interactions; they eventually sink, feeding the southward flowing lower limb of the AMOC^3^. In the subpolar North Atlantic (SPNA), properties like dissolved inorganic carbon –DIC–5,6,7,8, anthropogenic CO_2_ –C_ant_–9,10,11, trace metals like mercury^12^, and dissolved organic carbon –DOC–13 are transported to deeper layers as a result of water mass formation.

DOC is the largest pool of organic matter in the ocean^14^, thus playing an important role in ocean biogeochemistry. It is produced in the euphotic zone by biological activity and can be exported, i.e. moved to the deep ocean, by physical processes where it is remineralized. DOC export and its consumption in deep waters have implications for the removal of DIC from the surface, thus modifying air-sea CO_2_ exchanges. Oceanic DOC accumulated in the euphotic zone is redistributed horizontally by wind-driven surface circulation, then exported by (i) subduction in the subtropical gyres^14^,^15^,^16^, (ii) deep convection^17^,^18^,^19^ or (iii) overturning circulation at high latitudes^13^,^2^0. *Hansell et al.*^13^ estimated a DOC export in the whole Atlantic (72°S–63°N) via deep water formation of 0.086 Pg-C·yr^−1^, while *Carlson et al.*^20^ estimated it as 0.081 Pg-C·yr^−1^ for the North Atlantic (65°N–9°N).

We use an extended Optimum Multiparameter (eOMP) analysis as an objective tool to characterize the water mass DOC concentrations ([DOC]) along the OVIDE section (Fig. 1). The OVIDE section crosses the main currents of the eastern-SPNA gyre and some regions where water mass formation takes place. Water mass characterization allows us to estimate DOC’s horizontal transports across the section between 2002 and 2012 and their variability, even though DOC measurements were only performed in 2002^21^. Estimating carbon fluxes within water masses characterized for carbon content allows an assessment of the significance of the AMOC in carbon export and the impact of its variability in the marine carbon cycle. The main objectives of the present work are (i) to estimate the DOC transport and budget in the eastern-SPNA, and (ii) to disentangle the role of the AMOC in the DOC cycle.

## Methods

### OVIDE sampling program

**Figure 1:**
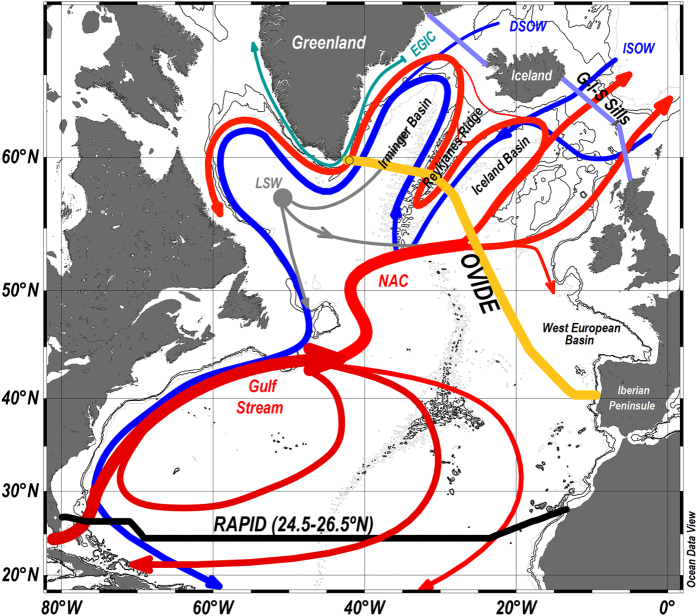
Map of the subpolar North Atlantic circulation with major topographical features included (the 1000, 1750 and 2500-m isobaths are plotted). Section tracks of the OVIDE (yellow thick line) and RAPID (24.5–26.5°N) (black thick line) cruises are indicated. The OVIDE box considered here lies between the OVIDE section and the Greenland-Iceland-Scotland (G-I-S) sills (purple line). Abbreviations for the main currents and water masses are as follows: DSOW = Denmark Strait Overflow Water, ISOW = Iceland-Scotland Overflow Water, LSW = Labrador Sea Water, EGIC = East Greenland Irminger Current, NAC = North Atlantic Current. The map was generated using Ocean Data View 4.7.1. Schlitzer,R., Ocean Data View, odv.awi.de, 2015. Schematic diagram of the large-scale circulation compiled from^3^,^23^,^30^,^49^.

**Figure 2:**
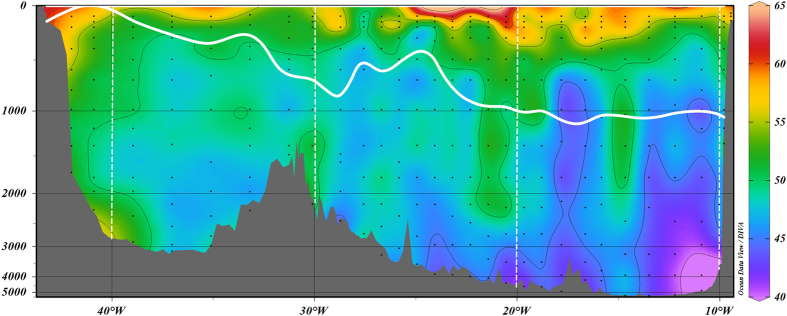
Dissolved organic carbon (DOC, in μmol·kg^–1^) vertical distribution along the OVIDE 2002 section from Greenland (left) to the Iberian Peninsula (right). Sampling points are indicated. Isopycnal σ_AMOC_ = 32.14 (potential density referred to 1000 dbar, solid white line) separating the upper and lower limbs of AMOC is also shown. The section was generated using Ocean Data View 4.7.1. Schlitzer,R., Ocean Data View, odv.awi.de, 2015. Note that the depth scale is not linear.

The OVIDE section, a high-resolution hydrographic survey from Portugal to Greenland, has been repeated biennially during spring-summer since 2002 (Fig. 1) (www.umr-lops.fr/en/Projets/Projets-actifs/OVIDE). In all cruises, high-quality measurements of standard tracers such as temperature, salinity (S), oxygen (O_2_) and nutrients were performed. Additionally, in 2002 (19 June–11 July), 30 stations were sampled for DOC (around 340 samples), and measured using a Shimadzu TOC5000 analyzer with a measurement error of ±0.7 μmol·kg^−1^ (Fig. 2)^21^. Samples for DOC (10 mL) were taken directly from the Niskin bottles without filtering process. The inclusion of particulate organic carbon from microorganisms in the samples is below the uncertainty. Besides, deeper than 1000 m there is no difference between filtered and unfiltered samples at the μmol·kg^−1^ resolution^22^. Therefore, we can assume that the total organic carbon (TOC) measured represents essentially the DOC reservoir. The accuracy was tested with reference materials provided by D.A. Hansell (University of Miami). Cruise data are available from the CCHDO (CLIVAR & Carbon Hydrographic Data Office) webpage (http://cchdo.ucsd.edu/).

### DOC water mass definitions by eOMP analysis

Here we use the eOMP analysis to infer the [DOC] along the OVIDE section for cruises when it was not measured. We first define the [DOC] of the water masses of the region. To do so, we used DOC data from the 2002 cruise –[*DOC*]^2002^– (Fig. 2) and the water mass structure of the 2002 cruise resulting from the eOMP analysis conducted by *García-Ibáñez et al.*^23^ where the distribution of 12 source water types (SWTs) are described. The eOMP analysis quantifies the proportions of the mixtures of the SWTs that contribute to a given water sample^24^,^25^. The SWTs were characterized by potential temperature (Θ), S, preformed O_2_ and nutrients. Mixing was solved by minimizing the residuals of the linear mixing equations in a non-negative least-square sense, where mass is stringently conserved and the contributions of the SWTs must be positive. The eOMP analysis was restricted to pressure ≥100 dbar to avoid the non-conservative behavior of Θ and S above the seasonal thermocline due to air-sea interactions.

The [DOC] of each SWT ([DOC]_*i*_) was solved by an inversion of the eOMP equations. We performed an inversion of a system of 340 equations (the number of samples below 100 dbar) and 12 unknowns ([DOC]_*i*_; Table 1):


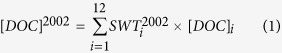


where *SWT*_*i*_^2002^ represents the proportion of each SWT for each sample of the OVIDE 2002 cruise with [*DOC*]^2002^. Surface seasonal variability is avoided by excluding samples from <100 dbar. The standard deviation of the residuals of Equation 1 is 2.6 μmol·kg^−1^, about 4 times the DOC measurement error, which is of the same order of fitting as that obtained for nutrients and O_2_ in the eOMP^23^. The uncertainties in the modeled [DOC] are fully taken into account when computing the error estimates for the DOC transports.

### DOC transports

Absolute transports across the OVIDE section were estimated using a linear box inverse model constrained by direct acoustic Doppler current profiler velocity measurements and by a net mass transport of 1 ± 3 Sv to the north^26^,^27^,^28^. Transport across OVIDE estimated by the inverse model has been previously evaluated^27^,^28^,^29^. The integration of AMOC transport in density levels, rather than in depth levels, better separates the upper and lower limbs of the overturning cell^30^ and avoids the partial cancellation that supposes the existence of opposed flows at overlapping depths when integrating AMOC transports in depth coordinates^28^. The boundary between the upper and lower limbs of the AMOC is the density level (σ_AMOC_) that corresponds to the maximum of the overturning streamfunction.

The water mass volume transports and structure for the upper and lower limbs of the AMOC have already been published by *García-Ibáñez et al.*^23^ for the period 2002–2010. Applying the same methodology, we solved the water mass volume transports for the 2012 cruise. Transports of DOC (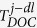) were integrated in density layers of 0.1 kg·m^−3^ (*j-dl*) and computed as the product of the volume transport (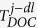), the [DOC]_*i*_, and the density (ρ) for each SWT:


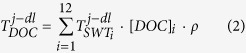


Since Equation 1 is restricted to ≥100 dbar, the contribution of the surface layer (<100 dbar) is accounted separately as:


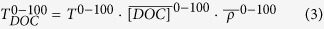


where *T*^ 0−100^ is the volume transport of the first 100 dbar, and 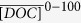 and 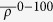 are the average [*DOC*]^2002^ and the average density in the upper 100 dbar, respectively.

Estimating transports in density levels allowed us to separate the DOC transport contributions of the upper and lower limbs of the AMOC. DOC transported by the lower limb of the AMOC was computed by integrating the DOC transports between σ_AMOC_ = 32.14 and the bottom. Similarly, DOC transported by the upper limb of the AMOC was computed as the sum of the surface layer contribution and the density-integrated DOC transport between 100 dbar and σ_AMOC_ = 32.14.

### DOC budget in the OVIDE box

We define the OVIDE box as the region bounded by the OVIDE section (southern boundary) and the Greenland-Iceland-Scotland (G-I-S) sills (northern boundary) (Fig. 1). The DOC budget in the OVIDE box is the net balance between DOC production/consumption due to biological activity and lateral advection due to ocean circulation through the OVIDE section and the G-I-S sills. Exchanges of water masses and DOC over the G-I-S sills, largely restricted by bottom topography and with a tight range of temporal variability, are available in the literature^31^,^32^,^33^,^34^ and can be also consulted in the Supplementary Information (Table S1). Regarding the linear interannual trend of the DOC content in the box, we evaluate the apparent oxygen utilization (AOU, difference between the measured O_2_ and its saturation concentration) as an indicator of DOC changes by cumulative respiration. The linear relationship between DOC and AOU^20^ can be as high as 0.88 in the northeastern North Atlantic^21^. The estimated AOU inventory for 2002–2012 shows no significant linear trend (see SI *text*), supporting that the interannual variation of the DOC content in the box is negligible.

Organic carbon inflows from rivers and rainwater could affect DOC budgets^35^. The amount of DOC discharged by the main rivers in the OVIDE box is 0.26 Tg-C·yr^−1 ^36. The global mean rate of atmospheric deposition of organic carbon because of rainwater flux has been estimated at 0.26 g-C·m^−2^·yr^−1^ 37 resulting in a net input of 2.0 Tg-C·yr^−1^ for the OVIDE box. Therefore, we neglected riverine and rainfall DOC inputs due to their small relevance compared to the lateral advection (~0.8%).

## Results and Discussion

### Water mass DOC characterization

Averaged [DOC] in the upper 100 dbar (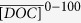) is 56.7 ± 0.6 μmol·kg^−1^, with values that range from 52.7 ± 0.2 μmol·kg^−1^ in the northernmost latitudes to 56.4 ± 0.6 μmol·kg^−1^ closer to the Iberian Peninsula, reaching >60 μmol·kg^−1^ in surface waters near 25°W (Fig. 2). The high [DOC] of the surface waters of the OVIDE section (40°N–60°N) present no meridional trend (p-level = 0.91), which contrasts with the latitudinal gradient previously reported for a zonal section in the North Atlantic^20^. Since the OVIDE section crosses North Atlantic Current (NAC) branches with waters of different ages, the DOC features observed in surface correspond to the variability of the mode waters due to their formation processes instead of a latitudinal gradient (Fig. 2). [DOC] decreases with depth to values <50 μmol·kg^−1^ in the deep waters, which are higher than those found in the deep waters of the South Atlantic and Pacific Oceans^13^. The lowest [DOC] is found in the Iberian Abyssal Plain (37.8 μmol·kg^−1^) due to the Antarctic Bottom Water influence^13^. North of 45°N (west of 18°W in Fig. 2), [DOC] is >45 μmol·kg^−1^ at 2000 m depth, resembling the distribution shown in *Hansell*^38^.

By SWTs (Table 1), the highest [DOC] is found in the ENACW_16_ (East North Atlantic Central Water of 16 °C; 59.0 ± 2.1 μmol·kg^−1^), in agreement with the high [DOC] of the subtropical systems^35^.

The DOC content of the Subpolar Mode Waters (SPMWs) is the result of the progressive renewal of the NAC waters during their cyclonic circulation in the Iceland and Irminger basins, which is evidenced by the [DOC] of SPMWs: the relatively old SPMW_8_ presents lower [DOC] (47.2 ± 1.1 μmol·kg^−1^) than the youngest IrSPMW (55.3 ± 1.1 μmol·kg^−1^), with the [DOC] of the intermediate stage, SPMW_7_, in between them (50.1 ± 1.1 μmol·kg^−1^). Thick layers with high [DOC] (52–54 μmol·kg^−1^) were also observed in the Irminger Basin^39^. At intermediate depths (1000–2500 m), Labrador Sea Water (LSW) is the dominant water mass of the section^23^, with a [DOC] of 48.1 ± 0.4 μmol·kg^−1^.

Both overflow waters, Iceland-Scotland Overflow Water (ISOW) and Denmark Strait Overflow Water (DSOW), contain relatively high [DOC] (Table 1). The [DOC] of ISOW is consistent with the mean [DOC] for the bathypelagic zone (1000–3000 m) at ~61°N (*Carlson et al.*^20^; Fig. 2c), typically dominated by ISOW^23^. The [DOC] of DSOW has been related to the high load of organic matter transported by the Arctic rivers^39^,^40^,^41^. This terrestrial influence should also affect the [DOC] of the Polar Intermediate Water, but its magnitude is conditioned by its reduced presence in the section due to its geographical constraint in the East Greenland slope^23^, which explains the high standard deviation of its [DOC] (48.4 ± 5.4 μmol·kg^−1^). The [DOC] of DSOW is responsible for the relative maximum located at the bottom of the west Irminger Basin (>50 μmol·kg^−1^), flowing along the East Greenland slope (west of 40°W) (Fig. 2). The [DOC] for the overflows are ~5 μmol·kg^−1^ lower than those found at the G-I-S sills^34^, consistent with the expected decrease of their [DOC] due to the entrainment processes occurring between the G-I-S sills and the OVIDE section. Conversely, North Atlantic waters flowing northward through the G-I-S sills carry similar or slightly higher [DOC] than observed in the OVIDE section, suggesting a balance between DOC production and respiration. The [DOC] of the lower North East Atlantic Deep Water is the lowest of the water masses (42.1 ± 0.6 μmol·kg^−1^, Table 1), in agreement with the values found in deep and bottom layers at 24.5°N where this water mass dominates^35^.

Our results from the SWT-DOC characterization are comparable with those of *Álvarez-Salgado et al.*^21^, who also used an OMP-based methodology but with a different water mass setting. The SWTs proposed by *García-Ibáñez et al.*^23^ for the eastern-SPNA allowed us to reach a better [*DOC*]_*i*_ adjustment (*r*^*2*^ = 0.68 vs 0.62) with a lower standard deviation of the residuals (SDR = 2.6 vs 3.1). The spatial distributions of modeled [DOC] resulting from the eOMP analysis (Fig. S2) reflects the expected DOC distribution in the North Atlantic, and present interannual variability due to the interannual variability in the observed water mass properties that are modeled as varying proportions of the SWTs. Furthermore, the [*DOC*]_*i*_ reached through our methodology agree with [DOC] previously reported. For example, *Amon et al.*^39^ reported a mean [DOC] of around 53 μmol·kg^−1^ for the Irminger Basin for samples taken in September–October 1998, in agreement with the values reported here. Besides, the good agreement between measured and modeled [DOC] in a meridional section along 20°W held in 2013, included the OVIDE box, (see SI *text*) supports the assumption of little interannual variability in the DOC content of each SWT ([*DOC*]_*i*_).

### DOC transport across the OVIDE section

**Figure 3:**
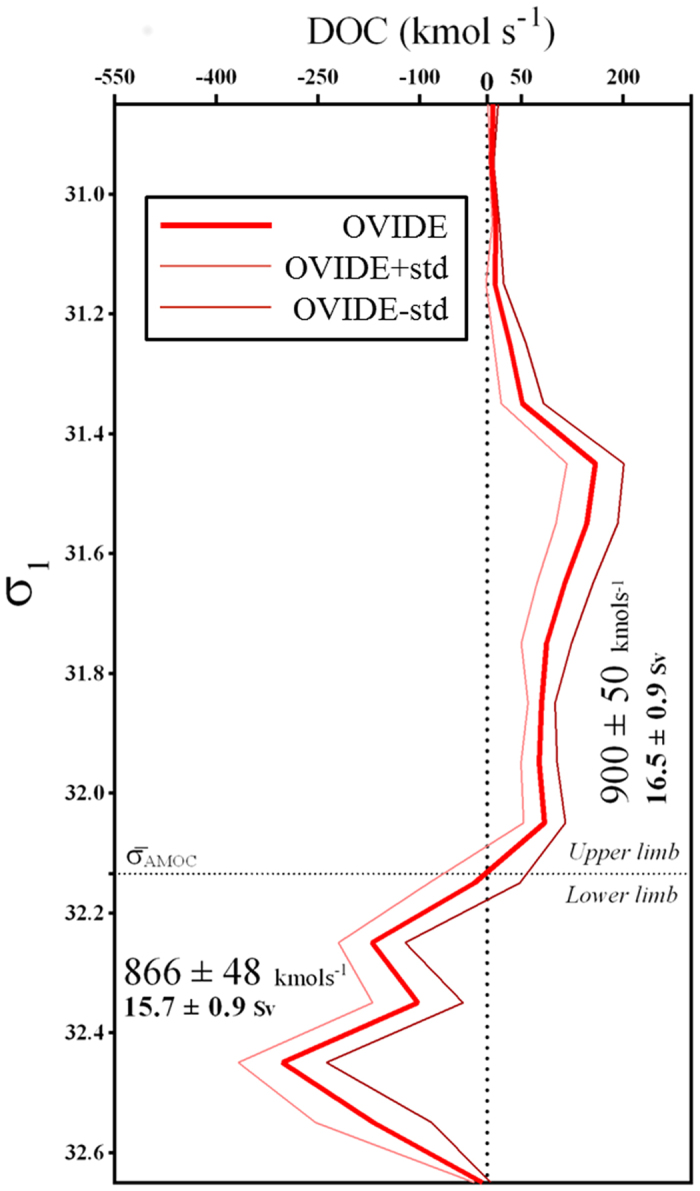
Average DOC transports (kmol·s^−1^, kilomoles by second) for period 2002–2012 (±standard deviation) integrated in density (σ_1_) layers with a 0.1 kg·m^−3^ resolution across the OVIDE section. Positive (negative) transports correspond to northward (southward) flow. The dashed horizontal line represents σ_AMOC_ = 32.14, the mean density boundary between the upper and lower limbs of the AMOC for the period 2002–2012. Net DOC transports in kmol·s^−1^ and volume transports in Sv (1 Sv = 10^6^·m^3^·s^−1^) are also presented.

Following *Lherminier et al.*^26^, DOC transports were integrated in density levels to better represent the circulation and the AMOC in the eastern-SPNA. Figure 3 shows the averaged profile of DOC transport for the six cruises (2002–2012) integrated from Greenland to Portugal and plotted by density layers. Integrated DOC transport resembles the vertical profile of the overturning circulation, with the classical two layers of transports in opposite directions. This profile resembles other tracer profiles such as heat and C_ant_^10^,^1^1, with a northward flowing upper layer and a southward flowing lower layer. The similarity is driven by the circulation, but C_ant_ and heat exhibit surface-intensified profiles while DOC is more balanced. The relative strength of the lower versus upper limb property transport is determined by the mean vertical gradient of the property; in terms of heat the lower limb’s role is relatively small, but for DOC, with a small vertical gradient, there is greater similarity between the upper and lower limb transports. A similar DOC transport profile was found in 24.5°N with northward volume transports in upper layers and southward volume transport in deeper layers^35^.

**Figure 4:**
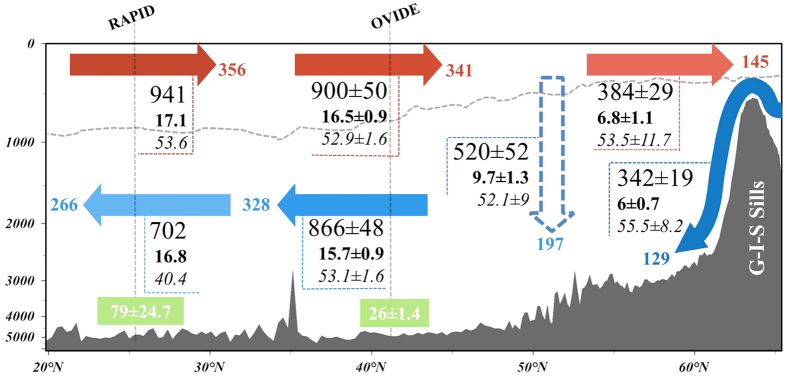
Mean DOC budget in the North Atlantic (from 24.5°N to 65°N) for the period 2002–2012. Schematic representation of the DOC transport (kmol·s^−1^, large font; and in Tg-C·yr^−1^ in color at the arrows tips), volume transport (Sv, bold numbers below DOC transport) and mean [DOC] (μmol·kg^−1^, italicized numbers below volume transport) between the RAPID section (left), the OVIDE section (middle) and the G-I-S sills (right). Values inside the green boxes represent the mean apparent oxygen utilization (in μmol·Kg^−1^) in the lower limb. Dashed blue arrow represents the downward export inferred from the observed values (solid arrows). Associated uncertainties (±) are depicted when available. The dashed line represents the separation between upper and lower AMOC limbs.

Southward transport of DOC across the section in the lower limb of the AMOC is 866 ± 48 kmol·s^−1^, consisting of two peaks: a lighter one (σ_1_ = 32.25) related to IrSPMW and a denser and stronger one related to LSW and the overflows (DSOW and ISOW). DOC transported by the upper limb of the AMOC, 900 ± 50 kmol·s^−1^, is dominated by the North Atlantic Central Waters. The mean velocity-weighted [DOC] advected northward in the upper limb of the AMOC across the OVIDE section ([DOC]_upper_) is 52.9 ± 1.6 μmol·kg^−1^, while the mean velocity-weighted [DOC] advected southward in the lower limb ([DOC]_lower_) is 53.1 ± 1.6 μmol·kg^−1^ (Fig. 4).

### DOC circulation in the eastern Subpolar North Atlantic

DOC transports across the OVIDE box are summarized in Fig. 4. The net DOC transport of 34 ± 8 kmol·s^−1^ (13 Tg-C·yr^−1^) at OVIDE was computed as the difference between the DOC transports by the upper and lower limbs of the AMOC. This net transport is associated with a net northward transport of 0.8 Sv across the section^8^. Through the G-I-S sills, 384 ± 29 kmol·s^−1^ of DOC are transported northward in the upper limb of the AMOC, while 342 ± 19 kmol·s^−1^ are flowing southward as overflow waters, resulting in a net northward DOC transport of 42 ± 33 kmol·s^−1^ (16 Tg-C·yr^−1^) across the sills. The high overturning circulation inside the eastern-SPNA (9.7 ± 1.3 Sv), with an average [DOC] of 52.1 ± 9 μmol·kg^−1^, results in a downward DOC transport of 520 ± 52 kmol·s^−1^ (197 Tg-C·y^−1^). In the OVIDE box, the difference between the net DOC transport across the sills (42 ± 33 kmol·s^−1^) and that across OVIDE (34 ± 8 kmol·s^−1^) is close to zero (8 ± 77 kmol·s^−1^). This balance contrasts with the DIC fixation of ~106 kmol·s^−1^ determined in the OVIDE box via an inorganic nutrient budget^42^, suggesting that the fate of DIC fixation should be vertical export of biogenic particles instead of an accumulation of DOC. DIC imbalance is also supported by large particulate organic carbon fluxes to the ocean interior occurring at 2000 m horizon in the Irminger and Iceland Basins^43^.

Despite modification of the thermohaline properties of the water masses due to air-sea interaction, the average [DOC] along the AMOC pathways between the G-I-S sills and the OVIDE section show no substantial differences (Fig. 4), suggesting a relatively conservative DOC circulation. The relatively high velocity-weighted [DOC] of the lower limb of the AMOC is due to three factors: (i) the important contribution of DSOW, (ii) the arrival of IrSPMW transported by the strong EGIC^3^,^29^, and (iii) the fact that the deep waters with low [DOC] appear mainly southeast of the NAC (Fig. 2), where their net volume transport is close to zero, which greatly attenuates their contribution to the velocity-weighted [DOC] of the lower limb of the AMOC.

### DOC exchanges between subtropical and subpolar gyres

Full-depth DOC data for the 24.5–26.5°N section have been published^35^ with volume transport data for the WOCE-A05 cruise in January/February 1998. We reconstructed the DOC transports using the RAPID-MOC time series^44^ for the period 2004–2014 and the DOC transports reported by *Hansell et al.*^35^. This method is different from that used for OVIDE. Combining velocity-weighted [DOC] along the latitude 24.5–26.5°N^35^ with volume transport data from RAPID (www.rapid.ac.uk/rapidmoc), DOC transports have been calculated (see SI *text* for calculation details) to allow comparison between subtropical latitudes and the OVIDE section. At RAPID, the DOC circulation is characterized by an upper-limb northward transport of 941 kmol·s^−1^ (17.1 Sv) and a lower-limb southward transport of 702 kmol·s^−1^ (16.8 Sv), resulting in a net northward DOC transport of 239 kmol·s^−1^ (~90 Tg-C·yr^−1^). This net DOC transport is quite similar to the 81 Tg-C·yr^−1^ previously reported by *Carlson et al.*^20^ for the North Atlantic, computed as the product of the [DOC] gradient along the ocean ventilation path and the ventilation rate (AMOC term).

The velocity-weighted [DOC] of the upper limb of the AMOC at RAPID (53.6 μmol·kg^−1^) is very similar to that found at the OVIDE section. However, a decrease in the velocity-weighted [DOC] of the lower-limb of the AMOC of 12.7 ± 1.2 μmol·kg^−1^ is observed between OVIDE and RAPID (40.4 μmol·kg^−1^). This DOC consumption results in a decrease in the DOC transport of the lower limb of the AMOC of 164 kmol·s^−1^ (~62 Tg-C·yr^−1^) between OVIDE and RAPID, which represents 72% of the DOC export of 86 Tg-C·yr^−1^ for the whole Atlantic^13^. Therefore, we found that most of the DOC remineralization occurs in the lower limb of the AMOC in contrast with results from *Carlson et al.*^20^, who located the DOC remineralization almost equally among the upper and lower limbs of the AMOC. The differences between estimates may be explained by the criteria used to distinguish the upper and lower limbs of the AMOC. The separation based on the density levels that maximizes AMOC transport^26^ applied in our study is quite different to the separation between the main thermocline versus North Atlantic Deep Water (NADW) ventilation rate applied by *Carlson et al.*^20^. By applying the density separation criteria, the important contribution of IrSPMW to the DOC transport is assigned to the lower limb of the AMOC and not shared between both limbs. In addition, the contribution of DSOW in the lower limb is not accounted for in *Carlson et al.*^20^ because it was not sampled. Assuming a level of ~40 μmol·kg^−1^ for the deep-water refractory [DOC], which has an estimated lifetime of 16,000 years^38^, we interpret the strong DOC decrease in the North Atlantic deep waters as a sign of the bioavailability of the DOC exported from the OVIDE box to the subtropical latitudes. This downstream DOC consumption at decadal time-scale is contributing to the CO_2_ production in deep waters^13^,^20^.

The relationship between DOC and AOU gradients could give insights about the origin of the differences found between OVIDE and RAPID. In our data, the ratio of ∆DOC/∆AOU-C_eq_ (converted to carbon equivalents with the molar ratio ∆C/∆O_2_ = −0.72^45^) is 33 ± 6%. This ratio is slightly above the 5–29% found by *Carlson et al.*^20^ for the same region. The enhanced contribution of DOC oxidation to oxygen consumption could be due to the proportion of non-refractory DOC and the relatively young age of the water masses^46^.

We are aware that the assumption of no seasonal variability deserves a close justification, even more so in the surface layer where biological activity could result in a seasonal variation of the [DOC] between winter lows of 42 μmol·kg^−1^ to summer highs of 65–70 μmol·kg^−1^^14^. However, the seasonal [DOC] range in the surface layer of the eastern North Atlantic is only ± 5 μmol·kg^−1^ 47. The DOC transported northward by the surface layer (0–100 dbar) at OVIDE section (76 kmol·s^−1^) represents ~9% of the DOC transported by the upper limb of the AMOC (Table S2). The surface layer (0–100 dbar) is where the seasonal [DOC] variability is most relevant. Based on our methodology, a 1 μmol·kg^−1^ perturbation of the [DOC] in the first 100 dbar results in a perturbation of 1.4 kmol·s^−1^ of the DOC transport. So the seasonal variability of the northward DOC transport would be ± 7 kmol·s^−1^. This would be a minor correction to the DOC transport at OVIDE. In addition, the seasonal [DOC] variability at OVIDE is expected to co-vary with that in the G-I-S sills, so that both lateral boundaries of the OVIDE box would be affected in the same way by the DOC seasonal changes of the surface layer. Therefore, neglecting seasonal variability in the surface layer does not generate any seasonal bias in the net DOC budget.

## Conclusions

A central characteristic of the DOC cycle in the eastern-SPNA is its export from the upper to the lower limbs of the AMOC. The relatively fast vertical transport of DOC contributes to carbon sequestration, analogous to the vertical transport of DIC. Nordic overflows are also adding important quantities of DOC to North Atlantic deep waters. DOC injected to the deep layers is more labile than the majority of the deep DOC observed in the South Atlantic and Pacific Oceans, so it is more susceptible to remineralization. In the North Atlantic, DOC consumption of 62 Tg-C·yr^−1^ taking place in the lower limb of the AMOC (bathypelagic and mesopelagic layers) between subpolar and subtropical latitudes consumes 72% of the DOC exported by the whole Atlantic Ocean.

This outcome implies that much of the net DOC exported with the overturning circulation in the eastern-SPNA, the major source of new DOC in the deep global ocean, is remineralized within decades, thus impacting deep microbial and dissolved organic matter compositional dynamics. DOC downward export due to overturning circulation acts as a carbon sink and represents a considerable contribution to CO_2_ sequestration. Given the atmospheric CO_2_ uptake of 0.20 Pg-C·yr^−1^ in the area^48^, the carbon sequestration mediated by DOC would represent ~30% of the total North Atlantic CO_2_ sink. Therefore, this mechanism has to be accounted for in current ocean carbon models and in studies carried out by the biogeochemical and microbiological communities. When DOC advection in the OVIDE box is considered, the eastern-SPNA presents a DOC balance, suggesting that DOC production is balanced by its removal.

The methodology applied here, coupling well-solved water mass transport along transoceanic sections with high-quality [DOC] measurements, could solve the problem of too few DOC data throughout the world oceans, and the North Atlantic in particular. However, more direct observations to test this assessment are required.

## Additional Information

**How to cite this article**: Fontela, M. *et al.* Dissolved Organic Carbon in the North Atlantic Meridional Overturning Circulation. *Sci. Rep.* **6**, 26931; doi: 10.1038/srep26931 (2016).

## Supplementary Material

Supplementary Information

## Figures and Tables

**Table 1:** Potential temperature (Θ), salinity (S) and dissolved organic carbon (DOC) of each source water type* considered here with their corresponding standard deviations.

	Θ (°C)	S	DOC (μmol·kg^−1^)
ENACW_16_	16.00 ± 0.13	36.20 ± 0.02	59.0 ± 2.1
ENACW_12_	12.30 ± 0.18	35.66 ± 0.03	55.4 ± 0.5
MW	11.7 ± 0.2	36.500 ± 0.011	45.1 ± 1.1
SAIW	6.0 ± 0.2	34.70 ± 0.03	51.7 ± 1.0
SPMW_8_	8.00 ± 0.11	35.230 ± 0.016	47.2 ± 1.1
SPMW_7_	7.07 ± 0.07	35.160 ± 0.006	50.2 ± 1.1
IrSPMW	5.00 ± 0.02	35.014 ± 0.013	55.3 ± 1.1
LSW	3.00 ± 0.19	34.87 ± 0.02	48.1 ± 0.4
ISOW	2.60 ± 0.08	34.980 ± 0.003	48.4 ± 1.2
DSOW	1.30 ± 0.06	34.905 ± 0.006	51.8 ± 1.9
PIW	0.0 ± 0.2	34.65 ± 0.03	48.4 ± 5.4
NEADW_L_	1.98 ± 0.03	34.895 ± 0.003	42.1 ± 0.6
*r*^2^			0.68
SDR			2.6
SDR/ε			3.7

Correlation coefficient (r^2^) between observed and estimated DOC is given together with the standard deviation of the residuals (SDR) and the SDR/ε ratios, with ε being the DOC measurement error.

^*^ENACW_16_ and ENACW_12_: East North Atlantic Central Waters; MW: Mediterranean Water; SAIW: Subarctic Intermediate Water; SPMW_8_ and SPMW_7_: Subpolar Mode Waters of the Iceland Basin and IrSPMW of the Irminger Basin; LSW: Labrador Sea Water; ISOW: Iceland-Scotland Overflow Water; DSOW: Denmark Strait Overflow Water; PIW: Polar Intermediate Water; and NEADW_L_: lower North East Atlantic Deep Water.
