# Supplementary material for: Dissolved Organic Carbon in the North Atlantic Meridional Overturning Circulation
Source: Sci Rep. 2016 May 31;6:26931. doi: 10.1038/srep26931 (PMC4886255; doi:10.1038/srep26931)
Supplement: Supplementary Information [file srep26931-s1.pdf]

Supplementary Information (SI) of:

Dissolved Organic Carbon in the North Atlantic Meridional Overturning Circulation

Marcos Fontela[a], Maribel I. García-Ibáñez[a], Dennis A. Hansell[b], Herlé Mercier[c], Fiz F. Pérez[a]

[a] Instituto de Investigaciones Marinas, IIM-CSIC, 36208 Vigo, Spain.

[b] Rosenstiel School of Marine and Atmospheric Science, RSMAS/OCE University of Miami, Miami, Florida, USA.

[c] CNRS, Laboratoire de Physique des Océans, UMR 6523 CNRS/Ifremer/IRD/UBO, Ifremer Centre de Brest, Plouzané, France.

SI Text

Specifications of the OMP analysis An extended Optimum Multiparameter (eOMP) analysis[1] was used to solve the water mass structure of the OVIDE section. OMP analyses are based on the premise that the water mass fractions that constitute a sample can be reproduced by an appropriate mixture of some well-known end-member water types, which are characterized by water mass tracers like Θ and S. OMP analyses obtain the water mass fractions (X_i) by solving a system of linear equations by minimization through a non-negative least square method. Each equation of the system is weighted in relation to the accuracy of the measured property. The main difference between classical (cOMP)[2] and extended OMP analyses is that the latter includes both conservative and non-conservative variables. We constrained the OMP analysis to the water samples with pressure ≥100 dbar to avoid the non-conservative behavior of Θ and S in the surface layer due to air-sea interactions after the last maximum of winter convection[3]. The OMP has been successfully used in previous studies with similar needs for solving water mass mixing[3–5]. The system of equations in the first step, the cOMP based on conservative variables, remains as follows:

$$\sum_{i=1}^{n} X_i * \theta_i^{SWT} = \theta^{sample} + R_\theta$$

$$\sum_{i=1}^{n} X_i * S_i^{SWT} = S^{sample} + R_S$$

$$\sum_{i=1}^{n} X_i * SiO_{2i}^{SWT} = SiO_2^{sample} + R_{SiO_2}$$

$$\sum_{i=1}^{n} X_i * NO_i^{SWT} = NO^{sample} + R_{NO}$$

$$\sum_{i=1}^{n} X_i * PO_i^{SWT} = PO^{sample} + R_{PO}$$

$$\sum_{i=1}^{n} X_i = 1 + R_{mass}$$

where *Rp* is the residual of each property *p* (Θ, S, SiO_2, NO=10.5*NO_3+O_2 and PO=175*PO_4+O_2 [6–8]) measured (p^{sample}) that the OMP tries to minimize and P_i^{SWT} is the property of each SWT_i. The last equation accounts for the mass conservation.

The cOMP analysis is solved for each mixing figure. The mixing figures are groups of SWTs that are susceptible to mix together, and are set considering the vertical characteristics and/or dynamics of the SWTs in the region of study. The analysis is applied to assign the mixing figure where the water sample presents the lowest residuals.

Using the same set-up as the cOMP, an eOMP analysis is solved also considering non-conservative variables (SiO_2, NO_3, PO_4 and O_2). A new unknown has to be considered, ΔO, which refers to changes in O_2 due to the remineralization of the organic matter.

$$\sum_{i=1}^{n} X_i * \theta_i^{SWT} = \theta^{sample} + R_\theta$$

$$\sum_{i=1}^{n} X_i * S_i^{SWT} = S^{sample} + R_S$$

$$\sum_{i=1}^{n} X_i * SiO_{2i}^{SWT} + \Delta O / r_{Si} = SiO_2^{sample} + R_{SiO_2}$$

$$\sum_{i=1}^{n} X_i * O_{2i}^{0SWT} - \Delta O = O_2^{sample} + R_{O_2}$$

$$\sum_{i=1}^{n} X_i * NO_{3i}^{0SWT} + \Delta O / r_N = NO_3^{sample} + R_{NO_3}$$

$$\sum_{i=1}^{n} X_i * PO_{4i}^{0SWT} + \Delta O / r_P = PO_4^{sample} + R_{PO_4}$$

$$\sum_{i=1}^{n} X_i = 1 + R_{mass}$$

where R_{SiO2} is 12, R_{NO3} is 10.5 and R_{PO4} is 175 [7,8]. The cOMP analysis selects the mixing figure based on conservative water mass tracers; once the mixing figures are selected, the estimates of the X_i are given by the eOMP analysis, which takes into account the effect of the biology in the measured variables. The methodology has been contrasted with available [DOC] data from a section inside the OVIDE box in 2013 (Leg 1 of A16N)[9]. We compare the measured [DOC] (487 samples) with the reconstructed [DOC] result of the combination of the water mass proportions of the A16N section ($X_i^{\mathrm{A16N\_2013}}$) with the source water types $\left[DOC\right]_i$ of Table 1 through the equation $\left[DOC\right]^{\mathrm{A16N\_2013}} = \sum_{i=1}^{12} SWT_i^{\mathrm{A16N\_2013}} \times \left[DOC\right]_i$. The correlation coefficient (r^2) between the measured and reconstructed [DOC] is 0.75, with a mean difference of 2.3±1.9 μmol·kg^{-1}, which is inside the uncertainty of the measurements.

Time evolution of the DOC content. To evaluate if the time derivative of DOC storage in the OVIDE box is negligible, we evaluated the inventory of apparent oxygen utilization (AOU) in the OVIDE box from 2002 to 2012 as a proxy of the DOC content (Fig. S1). The range of interannual variation in AOU is tightly constrained around ~124 μmol·kg^{-1} between 2002 and 2012, with a standard deviation as low as

±1.2 µmol·kg^{-1}. Therefore, the assumption of no considerable differences in the interannual variability of DOC transport is also supported by the oxygen that have been respired.

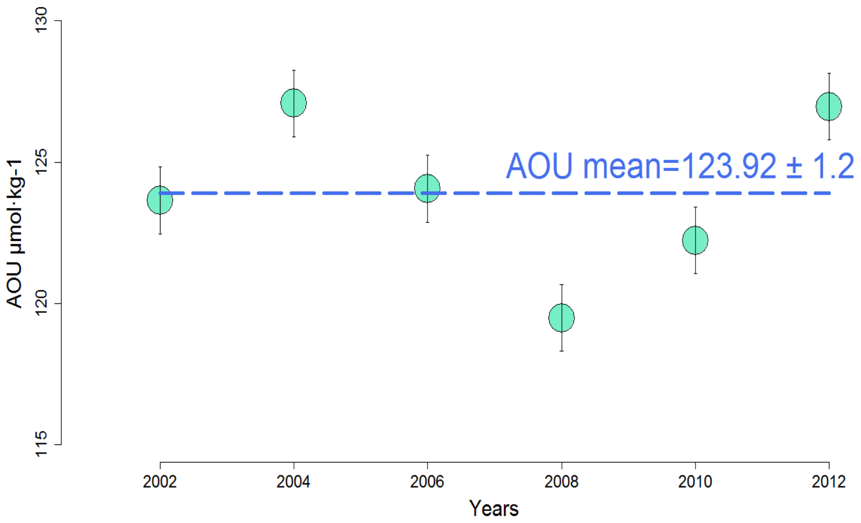

Figure S1. Apparent Oxygen Utilization (AOU, in µmol·kg^{-1}, ± standard deviation) at OVIDE line for each of the six cruises (2002–2012).

Error calculation. Assuming that the OVIDE cruises are repetitions, i.e. they had been performed equally and represent a similar oceanographic behavior, the error in the estimate of the DOC transport can be calculated simply as follows:

$$errorT_{DOC_{OVIDE}} = \sqrt{\frac{std}{n}}$$

where *std* is the standard deviation of the computed transports and *n* is the number of cruises between 2002 and 2012 (*n*=6).

Analytical computations of errors were performed at the G-I-S sills ($errorT_{DOC_{SILLS}}$) by means of a perturbation method. Independent normally-distributed perturbations (*n*=100 for each input variable) were generated using as the standard deviation the published uncertainties in the [DOC] data.

Budget error quantities in the OVIDE box were also computed through:

$$BUDGETerror = \sqrt{errorT_{DOC_{OV}}{}^2 + errorT_{DOC_{sills}}{}^2 + errorDOC_{storage}{}^2}$$

where $errorDOC_{storage}$ is evaluate from the variability of the inventory of DOC in the OVIDE box from 2002 to 2012 using the stoichiometric relationship between AOU and carbon (AOU-C_{eq}) and the proportion of carbon respired from the DOC pool (estimated in the article as 33±6%).

Reconstruction of DOC transports at 24.5ºN during the RAPID period. To get a robust DOC transport for the RAPID period (2004–2014), we reconstructed the DOC transports at subtropical latitudes (24.5–26.5ºN) based on the work of Hansell et al.[9] and the data of the RAPID-MOC time series[11]. This is a different approach from that used for the OVIDE section. First, we computed velocity-weighted mean [DOC] for each layer ($[DOC]_{mean}$'98, in $\mu mol \cdot kg^{-1}$) using the volume transports (T_{1998}, in Sv; 1 Sv=10^6 $m^3 \cdot s^{-1}$) and the DOC transports (T_{1998}DOC, in $kmol \cdot s^{-1}$) of the 24.5ºN cruise in January/February 1998 reported by Hansell et al.[9] (their Table 1). To obtain the same water column separation used for the RAPID-MOC time series[11], we restructured Hansell et al.[9]'s data for the upper limb of the AMOC into three layers: Ekman, upper mid-ocean and Gulf Stream. Volume transports and DOC transports for the Ekman layer were taken from Hansell et al.[9]'s Figure 4(c and d). Volume transports and DOC transports for the upper mid-ocean layer were obtained by adding the surface and intermediate layers in Hansell et al.[9]'s Table 1 and then removing the transports associated to the Ekman layer. Finally, $[DOC]_{mean}$'98 was combined with the average volume transport in RAPID-MOC time series[11] (T_{RAPID}, in Sv) for the period between 1 April 2004 and 22 March 2014, thus obtaining the reconstructed DOC transports at subtropical latitudes (24.5–26.5ºN) (T_{RAPID}DOC, in $kmol \cdot s^{-1}$). All data required for these computations are given in the following table:

	T_{1998} (Sv)	T_{1998}DOC ($kmol \cdot s^{-1}$)	$[DOC]_{mean}$'98 ($\mu mol \cdot kg^{-1}$)	T_{RAPID} (Sv)	T_{RAPID}DOC ($kmol \cdot s^{-1}$)	
Ekman	2.72	190.2	67.9	3.57	250	941
Upper mid-ocean	-21.65	-1239.6	55.6	-17.90	-1025	

Gulf Stream	30.49	1661.3	53.1	31.40	1716.4	
Deep ocean	-15.86	-659.4	40.6	-17.80	-746.4	-702
Deeper than 5000m	4.26	187.1	42.5	1.02	44.7	

The RAPID/MOCHA/WBTS array is a collaborative effort supported through the UK Natural Environment Research Council (NERC) RAPID-WATCH program, the US National Science Foundation (NSF) Meridional Overturning Circulation Heat-flux Array project, and the US National Oceanographic and Atmospheric Administration (NOAA) Western Boundary Time Series project; and transports including error estimates were freely available at www.rapid.ac.uk/rapidmoc

Figure S2. Interannual variability of modeled DOC

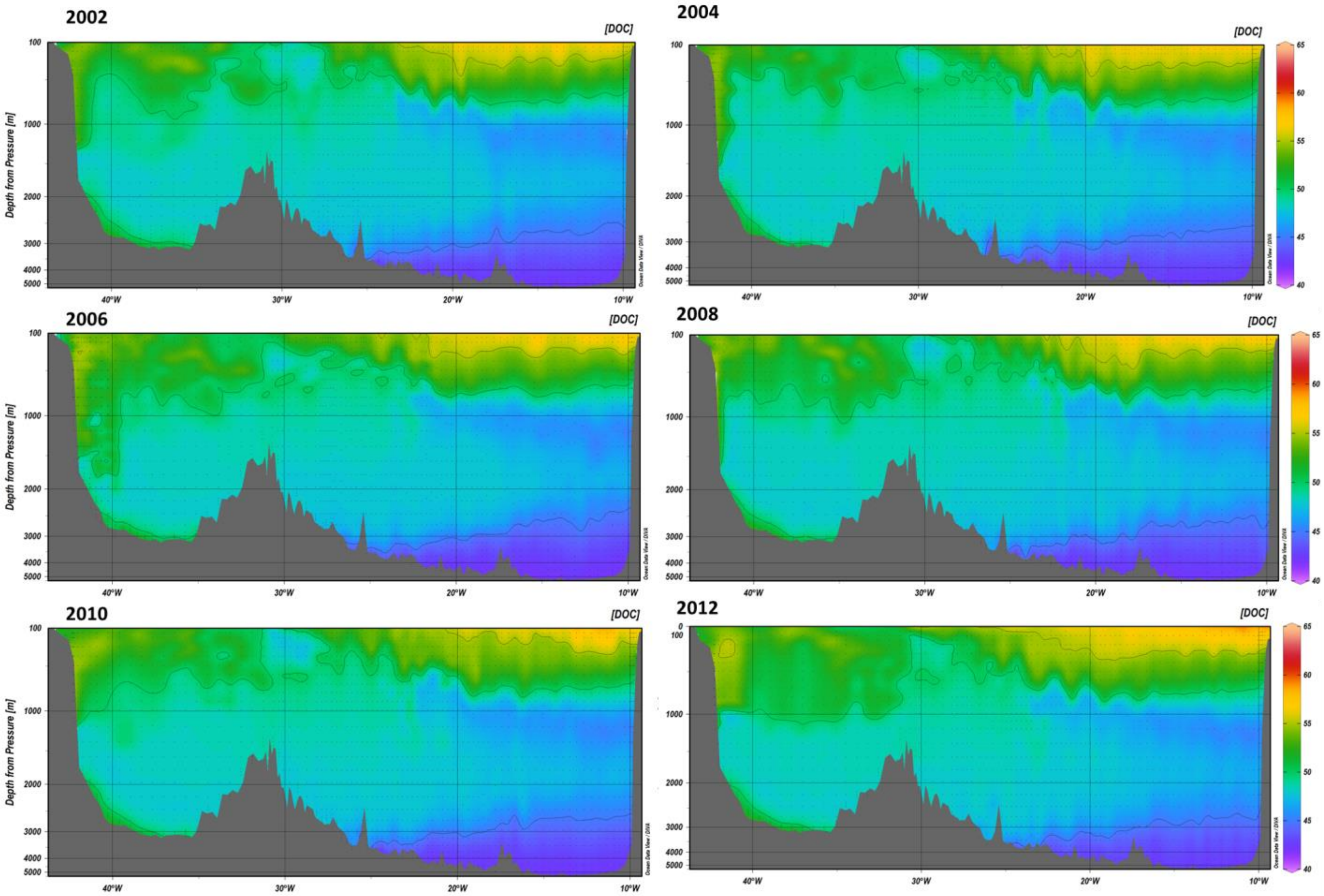

Figure S2. Dissolved organic carbon (DOC, in µmol·kg^{-1}) vertical distribution modeled along the OVIDE

section from Greenland (left) to the Iberian Peninsula (right) by combining water mass distributions with the source water types $\left[DOC\right]_i$ (see article Table 1) through the equation $\left[DOC\right]^{year} = \sum_{i=1}^{12} SWT_i^{year} \times \left[DOC\right]_i$. The sections were generated using Ocean Data View 4.7.1. Schlitzer,R., Ocean Data View, odv.awi.de, 2015. Note that the depth scale is not linear and the first hundred meters are excluded. The model is able to reproduce DOC changes in the sections between years based on the variability of the water mass contributions. In this way the model does not need the assumption of the time derivative of [DOC] being zero at OVIDE section. In addition, the model approach has the advantage of filtering any possible bias produced at single-station level. In the vertical distribution of [DOC] (Fig.2), there are some stations in the Iberian Abyssal Plain (east of 22ºW) showing a columnar vertical pattern that was not predicted by the eOMP, which means that it does not follow the water mass distributions.

Table S1. Water mass characterization at the Greenland-Iceland-Scotland (G-I-S) sills. Volume transport (in Sv; 1 Sv=10^6 $m^3 \cdot s^{-1}$) from Pérez et al.[11] (ENACW, East North Atlantic Central Water; MNACW, Modified North Atlantic Central Water; and NIIW, North-Iceland Irminger Water), Nilsson et al.[12] (PIW, Polar Intermediate Water), Macrander et al.[13] (DSOW, Denmark Strait Overflow Water), Hansen and Østerhus [14,15] (ISOW, Iceland-Scotland Overflow Water). Positive transports are northward. [DOC] (in $\mu mol \cdot kg^{-1}$) and density (in $kg \cdot m^{-3}$) are taken from Jeansson et al.[16]. The exchanges with the Nordic Seas are restricted by the G-I-S sill topography. The mean depth of the sill, around 500 m, limits the exchange of deep water with the North Atlantic. The only regions that allow relatively deep overflows are the Denmark Strait and the Faroe Bank Channel. Shallower overflows also occur across the Iceland-Faroe Ridge, a broad ridge with minimum depths of 300–500 m (deepening at the Faroese end), and the Wyville-Thomson Ridge between the Faroes and the Scotland shelf (depth ~600 m)[15]. This bathymetric restriction narrows the variability in annual circulation, so available literature data are well constrained.

Using data from the following table, we computed the T_{DOC} at the G-I-S sills as $T_{DOC}^{sills} = \sum_{i=1}^{6} T_{SWT_i}^{sills} \cdot [DOC]_i \cdot \overline{\rho}^{SWT_i}$.

Water mass	Volume transport (Sv)	Density ($kg \cdot m^{-3}$)	[DOC] ($\mu mol \cdot kg^{-1}$)
ENACW	3.85 ± 1	1027.3	58 ± 4
MNACW	3.85 ± 1	1027.4	58 ± 4
NIIW	0.8 ± 0.2	1027.6	59 ± 4
PIW	-1.8 ± 0.5	1027.4	70 ± 10
DSOW	-3 ± 0.3	1027.9	58 ± 6
ISOW	-3 ± 0.6	1028	53 ± 5

Table S2.Volume and DOC transports at OVIDE section. Volume (in Sv; 1 Sv=10^6 $m^3 \cdot s^{-1}$) and DOC transports (in $kmol \cdot s^{-1}$) at OVIDE section separated as surface layer (<100 dbar), the upper limb of the AMOC without the first 100 dbar, and the lower limb of the AMOC. The sum of the three components results in the net transport represented in the row labeled "Total". Northward transports are positive.

	2002		2004		2006	
	T_{Sv}	T_{DOC}	T_{Sv}	T_{DOC}	T_{Sv}	T_{DOC}
Surface layer	0.89	84	1.18	119	1.31	41
Upper limb >100 dbar	15.85	855	15.11	818	11.34	619
Lower limb	-16.5	-913	-16.35	-927	-11.25	-631
Total	0.24	26	-0.06	10	1.4	29

	2008		2010		2012		Mean	
	T_{Sv}	T_{DOC}	T_{Sv}	T_{DOC}	T_{Sv}	T_{DOC}	T_{Sv}	T_{DOC}
Surface layer	1.69	92	1.43	58	1.15	59	1.26	76
Upper limb >100 dbar	16.6	901	17.04	916	15.5	836	15.28	824
Lower limb	-17.37	-933	-17.23	-915	-15.71	-874	-15.73	-866
Total	0.92	60	1.24	59	0.94	21	0.8	34.2

Table S3. Mean water mass volume transports (in Sv; 1 Sv=10^6 $m^3 \cdot s^{-1}$) for the period 2002–2012 in the upper 100 dbar, the upper limb of the AMOC at depths >100 dbar, the lower limb of the AMOC and the whole water column (Total) at the OVIDE line. $ENACW_{16}$ and $ENACW_{12}$: East North Atlantic Central Waters; MW: Mediterranean Water; SAIW: Subarctic Intermediate Water; $SPMW_8$ and $SPMW_7$: Subpolar Mode Waters of the Iceland Basin and IrSPMW of the Irminger Basin; LSW: Labrador Sea Water; ISOW: Iceland-Scotland Overflow Water; DSOW: Denmark Strait Overflow Water; PIW: Polar Intermediate Water; and $NEADW_L$: lower North East Atlantic Deep Water. Positive transports are northward.

T_{Sv}	<100 dbar	Upper limb >100 dbar	Lower limb	Total
ENACW16	0.066	0.12	0	0.185
ENACW12	1.91	6.69	0.007	8.603
MW	0.00	0.074	0.005	0.08
SAIW	-0.30	3.99	-0.68	2.997
SPMW8	-0.002	1.74	0.28	2.022
SPMW7	0.71	1.78	0.47	2.961
IrSPMW	-1.04	0.2	-8.6	-9.44
LSW	0.013	0.68	-1.34	-0.65
ISOW	0	0.003	-2.71	-2.71
DSOW	0	0	-2.48	-2.48
PIW	-0.08	-0.036	-1.34	-1.45
$NEADW_L$	0	0.006	0.66	0.665

References

1. Poole, R. & Tomczak, M. Optimum multiparameter analysis of the water mass structure in the Atlantic Ocean thermocline. *Deep. Res. Part I Oceanogr. Res. Pap.* **46,** 1895–1921 (1999).

2. Tomczak, M. A multi-parameter extension of temperature/salinity diagram techniques for the analysis of non-isopycnal mixing. *Prog. Oceanogr.* **10,** 147–171 (1981).

3. García-Ibáñez, M. I. *et al.* Structure, transports and transformations of the water masses in the Atlantic Subpolar Gyre. *Prog. Oceanogr.* **135,** 18–36 (2015).

4. Vázquez-Rodríguez, M., Pérez, F. F., Velo, A., Ríos, A. F. & Mercier, H. Observed acidification trends in North Atlantic water masses. *Biogeosciences* **9,** 5217–5230 (2012).

5. Pardo, P. C., Pérez, F. F., Velo, A. & Gilcoto, M. Water masses distribution in the Southern Ocean: Improvement of an extended OMP (eOMP) analysis. *Prog. Oceanogr.* **103,** 92–105 (2012).

6. Broecker, W. S. 'NO', a conservative water-mass tracer. *Earth Planet. Sci. Lett.* **23,** 100–107 (1974).

7. Takahashi, T., Broecker, W. S. & Langer, S. Redfield ratio based on chemical data from isopycnal surfaces. *J. Geophys. Res.* **90,** 6907 (1985).

8. Anderson, L. A. & Sarmiento, J. L. Redfield ratios of remineralization determined by nutrient data analysis. *Global Biogeochem. Cycles* **8,** 65–80 (1994).

9. Baringer, M. *et al.* Carbon Dioxide, Hydrographic, and Chemical Data Obtained During the R/V Ronald H. Brown Cruise in the Atlantic Ocean on GO-SHIP/CLIVAR Repeat Hydrography Section A16N (Aug. 03 - Oct. 01, 2013). *Carbon Dioxide Information Analysis Center, Oak Ridge National Laboratory, US Department of Energy, Oak Ridge, Tennessee.* (2014). doi:10.3334/CDIAC/OTG.GOSHIP_A16N_2013

10. Hansell, D. A., Ducklow, H. W., Macdonald, A. M. & Baringer, M. O. Metabolic poise in the North Atlantic Ocean diagnosed from organic matter transports. *Limnol. Oceanogr.* **49,** 1084–1094 (2004).

11. McCarthy, G. D. *et al.* Measuring the Atlantic Meridional Overturning Circulation at 26°N. *Prog. Oceanogr.* **130,** 91–111 (2015).

12. Pérez, F. F. *et al.* Atlantic Ocean CO2 uptake reduced by weakening of the meridional overturning circulation. *Nat. Geosci.* **6,** 146–152 (2013).

13. Nilsson, J., Björk, G., Rudels, B., Winsor, P. & Torres, D. Liquid freshwater transport and Polar Surface Water characteristics in the East Greenland Current during the AO-02 Oden expedition. *Prog. Oceanogr.* **78,** 45–57 (2008).

14. Macrander, A., Send, U., Valdimarsson, H., Jónsson, S. & Käse, R. H. Interannual changes in the overflow from the Nordic Seas into the Atlantic Ocean through Denmark Strait. *Geophys. Res. Lett.* **32,** 1–4 (2005).

15. Hansen, B. & Østerhus, S. North Atlantic Nordic Seas Exchanges. *Prog. Oceanogr.* **45,** 109–208 (2000).

16. Hansen, B. & Østerhus, S. Faroe Bank Channel overflow 1995-2005. *Prog. Oceanogr.* **75,** 817–856 (2007).

17. Jeansson, E. *et al.* The Nordic Seas carbon budget: Sources, sinks, and uncertainties. *Global Biogeochem. Cycles* **25,** GB4010 (2011).
